# Inhibition of Biogenic Amines Formation in Fermented Foods by the Addition of Cava Lees

**DOI:** 10.3389/fmicb.2021.818565

**Published:** 2022-01-28

**Authors:** Salvador Hernández-Macias, Alba Martín-Garcia, Núria Ferrer-Bustins, Oriol Comas-Basté, Montserrat Riu-Aumatell, Elvira López-Tamames, Anna Jofré, M. Luz Latorre-Moratalla, Sara Bover-Cid, M. Carmen Vidal-Carou

**Affiliations:** ^1^Departament de Nutrició, Ciències de l’Alimentació i Gastronomia, Facultat de Farmàcia i Ciències de l’Alimentació, Campus de l’Alimentació de Torribera, Universitat de Barcelona (UB), Santa Coloma de Gramenet, Spain; ^2^Institut de Recerca en Nutrició i Seguretat Alimentària (INSA⋅UB), Universitat de Barcelona (UB), Santa Coloma de Gramenet, Spain; ^3^Xarxa d’Innovació Alimentària (XIA), Barcelona, Spain; ^4^Programa de Funcionalitat i Seguretat Alimentàries, Institut de Recerca i Tecnologia Agroalimentàries (IRTA), Finca Camps i Armet s/n, Monells, Spain

**Keywords:** cava lees, food by-product, bread, fermented sausages, biogenic amines, cadaverine, putrescine, tyramine

## Abstract

Food safety can be compromised by some bioactive compounds such as biogenic amines that can be specially found in fermented foods due to the bacterial decarboxylation of some amino acids by fermentative or spoilage bacteria. Cava lees are a winery by-product rich in fiber and phenolic compounds and previous works have raised their revalorization from a food safety point of view. The aim of the current work was to investigate whether the use of cava lees can help to control biogenic amine formation in bread and fermented sausages. In bread, only very low levels of biogenic amines (putrescine, cadaverine, and/or spermidine) were found, whose content did not vary with the addition of different amounts of lees. However, the addition of lees in fermented sausages significantly reduced the formation of tyramine and cadaverine. In sausages spontaneously fermented and inoculated with *Salmonella spp.*, the presence of cadaverine and putrescine diminished by 62 and 78%, respectively, due to the addition of cava lees. The addition of cava lees phenolic extract also showed an anti-aminogenic effect (21% for cadaverine and 40% for putrescine), although in a lesser extent than cava lees. Cava lees and their phenolic extract were shown to be an effective strategy to control the undesirable accumulation of high levels of biogenic amines during the production of fermented products.

## Introduction

Food safety can be compromised not only by food-borne pathogens but also by some bioactive compounds of bacterial origin such as biogenic amines. These microbial metabolites (i.e., tyramine, histamine, putrescine, and cadaverine) are formed by a decarboxylation of their precursor amino acids and can be found in almost all types of food in a wide range of concentrations, which may vary even within the same type of product. Histamine and tyramine can have adverse health effects, such as histamine intoxication, histamine intolerance or hypertensive crises caused by the interaction between tyramine and monoamine oxidase inhibitor (MAOI) drugs (Latorre-Moratalla et al., 2017). In fact, histamine and histamine intoxication are one of the hazards and foodborne diseases annually included in report issued by the European Food Safety Authority (EFSA) and the European Centre for Disease Prevention and Control (ECDC) (European Food Safety Authority [EFSA] and European Centre for Disease Prevention and Control [ECDC], 2021). In addition, histamine can also be the causative agent of histamine intolerance, a disorder that appears in individuals sensitive to histamine levels generally tolerated by healthy population mainly as a consequence of a deficiency of diamine oxidase (DAO), an enzyme responsible for its intestinal degradation (Comas-Basté et al., 2020).

Besides the food safety issue, because the accumulation of biogenic amines in foods can be associated with the activity of spoilage bacteria, a high content of these compounds is considered a chemical marker of insufficient food freshness or poor hygiene in the manufacturing and/or food preservation processes (Comas-Basté et al., 2019). Additionally, fermented foods are also susceptible to accumulate significant levels of amines, not only associated with the activity of the undesired contaminant bacteria, but also with fermentative microorganisms (Latorre-Moratalla et al., 2017). In recent decades, hygienic improvements at all stages of the food chain, as well as the implementation of specific recommendations, such as the use of non-aminogenic starter cultures, have contributed to minimize the levels of these compounds in fermented products. However, current data on the occurrence of biogenic amines in this kind of products show that this area of food safety continues to be a challenge for the food industry. New strategies based on natural products are being developed and can complement or even replace traditional systems used to prevent or inhibit the growth of aminogenic microorganisms (Zhang et al., 2017).

Cava is a quality Spanish sparkling wine with Protected Designation of Origin Cava, made using the traditional method, which involves a second fermentation, aging and disgorging in the same bottle that reaches the consumer (BOE-A-1991-28079, 1991). Cava lees are a by-product from the production of this beverage consisting of plasmolyzed and inactive cells of *Saccharomyces cerevisiae* (Tudela et al., 2012). Lees are the second most generated by-product of the wine industry, and an estimated 300 tons of cava lees are produced in Spain per year (Hernández-Macias et al., 2021b). It has recently been pointed out that the addition of cava lees in the formulation of certain fermented foods can have positive effects regarding microbiological safety. In particular, their richness in fiber could promote the growth of fermentative bacteria and thus bring about a more rapid and greater reduction in *pH* (Hernández-Macias et al., 2021a). Moreover, the lower *pH* of cava lees and/or other compounds found in lees, such as polyphenols and organic acids, could also exert an antimicrobial effect against undesired foodborne microorganisms. A recent study reported that cava lees added to fermented sausages significantly inhibit the growth of *Salmonella* spp. and *Listeria monocytogenes* (Hernández-Macias et al., 2021b).

The aim of the current work was to investigate whether the use of cava lees can reduce biogenic amine formation in bread and fermented sausages. If confirmed, this revalorization strategy for cava lees would provide a new advantage from the food safety point of view, in addition to the control of food-borne pathogens. According to our knowledge, this is the first time that the applicability of cava lees to control the accumulation of biogenic amines in fermented foods have been studied.

## Materials and Methods

### Cava Lees and Cava Lees Phenolic Extract

Cava lees were provided by the winery Freixenet S.A. (Sant Sadurní d’Anoia, Spain). To remove any remaining cava, the wet lees were centrifuged at 18,000 × *g* for 10 min at 4°C. Subsequently, the lees were frozen (−80°C), lyophilized (Cryodos-50, Telstar, Terrassa, Spain), ground and stored in tubes protected from light and humidity until use. A lees phenolic extract (LPE) with a total phenolic content of 152.2 ± 3.5 mg GAE/g was obtained from the cava lees using the method described by Silva et al. (2018).

### Preparation of Bread

Bread was prepared using 500 g of wheat flour, 150 g of sourdough, 285 g of water, 4 g of lyophilized yeast (*Saccharomyces cerevesiae*) and 10 g of salt. Four batches of bread were made: without cava lees (control) and with three different amounts of cava lees (1, 2, and 5% w/w of flour). These percentages were selected for showing a growth promoting effect on fermentative lactic acid bacteria (LAB) of the sourdough with total counts determined using MRS medium at 30°C under aerobic conditions during 72 h. The sourdough was prepared following the procedure described by Wehrle and Arendt (1998). The ingredients were manually kneaded and then fermented for 2 h at room temperature. The fermented dough was baked at 200°C for 30 min. Each type of bread was made in triplicate.

### Preparation of Fermented Sausages

The meat batter was prepared by mixing minced pork lean and ground fat (8:2) through a 6 mm plate. The ground meat was then mixed with (g/kg): sodium chloride (25), dextrose (7), black pepper (3), sodium ascorbate (0.5), sodium nitrite (0.15), and potassium nitrate (0.15).

Firstly, two different batches were made: without (control) and with 5% (w/w) cava lees. According with previous studies, 5% of cava lees was the most effective amount to enhance the *in vitro* bacterial growth in MRS medium of different strains of starter cultures, such as *Latilactobacillus sakei*, *Lactiplantibacillus plantarum*, *Latilactobacillus curvatus*, *Lacticaseibacillus casei*, and *Limosilactobacillus fermentum* (Hernández-Macias et al., 2021a), as well as to inhibit the growth of pathogenic bacteria in fermented sausages (Hernández-Macias et al., 2021b). For each batch, 80 g portions of the prepared meat batter were vacuum stuffed into Tublin10 permeable plastic casings (Tub-Ex, Tass, Denmark), subjected to a fermentation process (2 days at 23°C) and subsequently ripened (19 days at 15°C). Each batch was made in triplicate.

In addition, the effect of cava lees on biogenic amine formation was also studied in more detail using samples of fermented sausage from a previous study, in which different strains of *Salmonella enterica* were inoculated to the meat batter at a final level of ca. 6 log_10_ CFU/g (same amount for each strain): CTC1003 (serotype London), CTC1756 (serotype Derby) and CCUG34136 (serotype Enteritidis, Type strain) (Hernández-Macias et al., 2021b). Portions of 80 g of the inoculated meat batter were stuffed into Tublin10 permeable plastic casings as previously described. Table 1 shows the different batches of fermented sausages considered in experiment 1, which evaluated the effect of adding 5% (w/w) cava lees, and in the experiment 2, with the addition of 0.3% of cava lees phenolic extract (LPE). The starter cultures used were the strains *L. sakei* (formerly *Lactobacillus sakei*) CTC494, producer of the bacteriocin sakacin K (Garriga et al., 2002), and *L. sakei* BAP 110.

**TABLE 1:** Batches of fermented sausages included in each experiment, formulated with or without 5% cava lees or 0.3% lees phenolic extract (LPE) and/or different strains of *L. sakei* (CTC494 or BAP110) as the starter culture.

Experiment	Batch	Ingredients	Starter culture strain^1^
1	C1	−	−
	L1	Cava lees	−
	C1 + CTC494	−	*L. sakei* CTC494^2^
	L1 + CTC494	Cava lees	*L. sakei* CTC494
2	C2	−	−
	E2	LPE	−
	C2 + CTC494	−	*L. sakei* CTC494
	E2 + CTC494	LPE	*L. sakei* CTC494
	C2 + BAP110	−	*L. sakei* BAP110
	E2 + BAP110	LPE	*L. sakei* BAP110

*^1^Batches without starter cultures (−) underwent spontaneous fermentation.*

*^2^L. sakei CTC494 produces the bacteriocin sakacin K (Garriga et al., 2002).*

The analyzed samples corresponded to the meat batter (time 0), 8–9 days of ripening (depending on the experiment) and the finished product after 21 days of ripening. Each batch was prepared in triplicate.

### Biogenic Amine Analysis and Bacterial Decarboxylase Activity

Twelve biogenic amines (octopamine, dopamine, tyramine, putrescine, serotonin, cadaverine, histamine, agmatine, β-phenylethylamine, tryptamine, spermidine, and spermine) were determined in bread (sourdough and baked bread) and fermented sausages (at different time points during fermentation and ripening by triplicate). The biogenic amine content in the cava lees was also analyzed. Ultra-high efficiency liquid chromatography coupled to fluorometric detection (UHPLC-FL) was used according to Latorre-Moratalla et al. (2009). Briefly, approximately 7 g of bread and 5 g of fermented sausage were mixed with 10 mL of 0.6 M perchloric acid for 20 min. Subsequently, the samples were centrifuged (18,000 × *g*, 4°C, 25 min) and the supernatant was collected in a 25 mL volumetric flask. This extraction process was repeated twice more, and the final volume of the extract was adjusted with 0.6 M perchloric acid. The samples were filtered through a 0.22 μm GHP filter. All determinations were done in triplicate.

Chromatographic separation of the biogenic amines was performed using an Acquity UPLC BEH C18 1.7 μm (2.1 mm × 50 mm) reversed phase column (Waters Corp., Milford, MA, United States), followed by an on-line post-column derivatization with *ortho*-phthaldehyde and a fluorometric detection (Ex: 340 nm and Em: 445 nm).

The aqueous content was determined (AOAC, 2000) and the biogenic amine content during the manufacturing of fermented sausages was expressed as dry matter (dm) to avoid the increase of concentration due to drying during sausage ripening.

The amino acid decarboxylase activity of the strains (*L. sakei* BAP110 and *Salmonella* spp.) was evaluated using the method described by Latorre-Moratalla et al. (2010). The absence of decarboxylase activity in *L. sakei* CTC494 was previously established by Bover-Cid et al. (2001).

### Statistical Analysis

To determine the statistical differences in biogenic amine contents between the different batches of fermented products, a one-way ANOVA and the Tukey HSD *post hoc* test were performed (SPSS software). Differences with *p* < 0.05 were considered statistically significant.

## Results and Discussion

### Effect of Cava Lees on the Biogenic Amine Content in Bread and Fermented Sausages

The biogenic amines detected in the sourdough and different batches of bread are shown in Table 2. According to Gänzle and Ripari (2016), sourdough contains fermenting yeast (*S. cerevisiae)* and LAB, mainly *Lactobacillu*s spp. and *Leuconostoc* spp., whose acidifying and proteolytic properties influence the aromatic quality and rheology of bread. Despite this considerable microbial activity, the sourdough was found to contain only low levels of putrescine and spermidine, both naturally present in flour and therefore coming from this ingredient (Karayigit et al., 2020).

**TABLE 2:** Biogenic amine contents (mean ± SD in mg/kg fresh matter) in sourdough, in baked bread with and without the addition of different amounts of cava lees (1, 2, and 5% [w/w of flour]), and in spontaneously fermented sausages with and without the addition of 5% (w/w) cava lees after 21 days of ripening.

	Tyramine	Putrescine	Cadaverine	Histamine	Spermidine	Spermine
Cava lees	nd	nd	nd	nd	10.5 ± 0.43	0.10 ± 0.03
**Bread**						
Sourdough	nd	2.02 ± 0.03	nd	nd	1.52 ± 0.01	nd
Bread	nd	3.79 ± 0.02^a^	nd	nd	4.28 ± 0.02^a^	nd
Bread + 1% cava lees	nd	4.21 ± 0.01^a^	2.87 ± 0.03^a^	nd	5.38 ± 0.01^a^	nd
Bread + 2% cava lees	nd	5.50 ± 0.04^a^	2.78 ± 0.00^a^	nd	4.72 ± 0.05^a^	nd
Bread + 5% cava lees	nd	3.73 ± 0.23^a^	2.93 ± 0.02^a^	nd	5.58 ± 0.04^a^	nd
**Fermented sausages**						
Fermented sausage	17.66 ± 0.46	10.70 ± 0.34^a^	39.78 ± 1.46^a^	nd	6.42 ± 0.03^a^	22.60 ± 0.47^a^
Fermented sausage + 5% cava lees	nd	11.04 ± 0.35^a^	09.35 ± 0.32*^b^*	nd	6.90 ± 0.09^a^	26.32 ± 1.49^a^

*nd: not detected (<0.05 mg/kg); Significant differences between control batches and lees supplemented batches are indicated by different superscript letters (p < 0.05).*

As in the sourdough, the only amines detected in bread made without cava lees were putrescine and spermidine, whose content did not change significantly due to the addition of cava lees, irrespectively of the amount used. Bread made with cava lees was also found to contain very little concentration of cadaverine, thus the lees supplementation hardly had a significant effect on its formation. Neither could the lees be the source of cadaverine, considering that the only amine they contained in a significant amount was spermidine.

The fact that no biogenic amines related to microbial activity were found in sourdough or bread could be due either to a lack of aminogenic capacity by fermentative strains of *S. cerevisiae* or LAB, or because the short fermentation that takes place for the preparation of the bread does not allow the formation of these compounds.

In contrast with other fermented products, relatively few data are available on biogenic amines in bread. Putrescine, cadaverine, histamine, and tyramine are the most frequently reported (Farkas and Hajós, 1998; Cohen et al., 2014; Diana et al., 2014). Quantitatively, the amine contents are usually low, normally below 10 mg/kg, without differences between bread prepared with starter cultures or sourdough (Diana et al., 2014). However, high contents of some of these amines have occasionally been described. For instance, Cohen et al. (2014) found high levels of putrescine and cadaverine in bread samples that gave off a penetrating odor, which was related to a bacterial spoilage of the product, though the spoilage microorganisms were not identified. In contrast, the high levels of tyramine reported by Diana et al. (2014) were not due to spoilage, but to the decarboxylase activity of a *Lactobacillus brevis* strain added as a starter culture. Future studies should consider the use of an amine-producing LAB strain as starter culture to better assess the capability of lees to reduce the formation of biogenic amine in bread.

The spontaneously fermented sausages, both with and without cava lees, contained tyramine, putrescine, and/or cadaverine (Table 2), amines that can be originated from bacterial decarboxylase activity and are typically found in this kind of products (Latorre-Moratalla et al., 2017). Spermine and spermidine, natural polyamines with a fundamentally physiological origin, were also detected, whose main source would have been the raw meat material. Spermine levels were higher than those of spermidine, as described for this type of food of animal origin (Vidal-Carou et al., 2014).

In the control batch, cadaverine was the dominant amine, representing 41% of the total amine content, followed by spermine (23%) and tyramine (18%), whereas putrescine and spermidine were present at the lowest amounts. The effect of cava lees in fermented sausages was only evaluated with the addition of 5% of lees, since this was the amount that showed an antimicrobial effect in this kind of product (Hernández-Macias et al., 2021b). In the same line, the addition of 5% of lees had an effect in reducing the content of amines. Specifically, the presence of lees completely prevented the formation of tyramine and significantly (*p* < 0.05) reduced cadaverine accumulation by more than 76% compared to the control batch.

Based on these promising results, the potential reductor effect of cava lees on biogenic amine formation during the production of fermented sausages was studied in more depth using different batches of fermented sausages from a previous study (Hernández-Macias et al., 2021b).

### Effect of Cava Lees on the Evolution of Biogenic Amines During the Production of Fermented Sausages Inoculated With Aminogenic Bacteria

Fermented sausages were inoculated with different strains of *Salmonella*, a bacterial genus generally linked with a high capacity to form biogenic amines. However, as aminogenic capacity varies among strains, it is necessary to carry out a case-by-case evaluation (Comas-Basté et al., 2019; Tabanelli, 2020). The three inoculated strains of *Salmonella* spp. displayed strong aminogenic activity *in vitro*, forming high levels of cadaverine and putrescine (2,500 and 2,300 mg/L, respectively), and moderate levels of histamine (200 mg/L); the strains were not able to form tyramine. The production of putrescine, cadaverine, and histamine is frequently associated with the activity of Gram-negative spoilage bacteria (*Pseudomonas* and *Enterobacteria*) and food-borne pathogenic bacteria (Suzzi and Gardini, 2003; Latorre-Moratalla et al., 2012; Kukleci et al., 2019). In a comparative study, Gokdogan et al. (2012) reported that *Salmonella* was among the pathogenic bacteria with the highest capacity to produce these amines.

Moreover, some batches were also inoculated with *L. sakei* strains used as starter cultures. Both *L. sakei* strains were amino acid decarboxylase-negative *in vitro*. Bover-Cid et al. (2001) previously demonstrated that *L. sakei* CTC494 cannot form biogenic amines, a capacity generally described as weak or absent in this specie (Latorre-Moratalla et al., 2012; Özogul and Hamed, 2018).

Biogenic amine content during sausages manufacture at different sampling points are referred to dry matter (dm) to avoid the concentration effect of the drying process. In the meat batter used in the manufacturing of fermented sausages for both experiments, spermine and spermidine were the only two amines detected, with contents of 89.2 ± 0.2 and 3.4 ± 0.1 mg/kg dm, respectively, which did not change significantly during the fermentation and ripening process in any of the batches. These results confirm the physiological origin of these polyamines from meat. Although the cava lees contained spermidine, the proportion in which the lees were added to the sausages (5%) was too low to create significant differences with the control batches.

**FIGURE 1:**
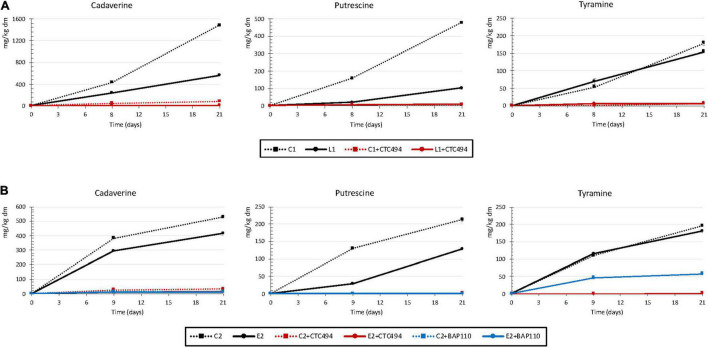
Contents of cadaverine, putrescine, and tyramine (mg/kg dry matter) in fermented sausages: **(A)** Spontaneous fermentation without (C1) or with (L1) the addition of 5% cava lees or fermentation with the starter culture *L. sakei* CTC494 without (C1 + CTC494) or with (L1 + CTC494) cava lees; **(B)** Spontaneous fermentation without (C2) or with (E2) the addition of 0.3% LPE or fermentation with the starter culture *L. sakei* CTC494 or *L. sakei* BAP110 without (C2 + CTC494 or C2 + BAP110) or with (E2 + CTC494 or E2 + BAP110) the LPE.

During the fermented sausage processing, three amines were detected, cadaverine, putrescine and tyramine, the concentrations varying considerably between experiments and/or batches (Figure 1). Physicochemical parameters and microbiological counts of all batches of fermented sausages were previously reported in Hernández-Macias et al. (2021b).

In the spontaneously fermented control sausages of experiment 1 (C1), cadaverine was the major amine, reaching levels of 1,484 mg/kg dm (Figure 1A). Significant amounts of putrescine were also formed throughout the production process, with levels of 479 mg/kg dm. The high contents of cadaverine and putrescine can presumably be attributed to the strong capacity of the inoculated *Salmonella* strains to synthesize these diamines. Both amines are commonly found in fermented meat products, although usually at relatively low levels (around 50 mg/kg, according to the risk assessment by EFSA) (European Food Safety Authority [EFSA], 2011). However, in some cases, their levels can increase dramatically (up to 1,500 mg/kg) attributable to contamination of raw materials with enterobacteria (Latorre-Moratalla et al., 2008; European Food Safety Authority [EFSA], 2011; Ruiz-Capillas and Herrero, 2019). The content of tyramine in C1 also increased progressively throughout the production process, although to a lesser extent than the other two amines (179 mg/kg dm). According to European Food Safety Authority [EFSA] (2011), the average tyramine value found in different European fermented sausages is about 130 mg/kg, but reaching a maximum of more than 1,700 mg/kg. Tyramine formation can be partially attributed to the activity of LAB, including enterococci and lactobacilli, some of them mainly responsible for fermentation, but also associated with contamination of raw materials (Anderegg et al., 2020). For this reason, tyramine tends to be the predominant and most characteristic amine of fermented meat products (Latorre-Moratalla et al., 2017). In the current work, the inoculation of sausages with *Salmonella* spp., which has a high capacity to produce cadaverine and putrescine but not tyramine, could explain that the contents of cadaverine and putrescine exceed those of tyramine.

The addition of cava lees (L1) only affected the formation of cadaverine and putrescine, with values in the finished product being 62 and 78% lower than in the control batch C1, respectively (*p* < 0.05). The markedly lower counts of *Salmonella* (up to 2.7 log_10_) found in fermented sausages formulated with cava lees (Hernández-Macias et al., 2021b) could explain the lower putrescine and cadaverine levels. Regarding tyramine, no significant differences were observed between batches without (C1) and with lees (L1). The fact that the LAB counts were not influenced by the addition of cava lees (Hernández-Macias et al., 2021b) could explain why this by-product supplementation had no effect on tyramine formation.

Finally, the formation of cadaverine, putrescine, and tyramine was inhibited in the batches added with the starter culture *L. sakei* CTC494 (Figure 1A), a highly competitive and non-aminogenic strain, resulting in fermented sausages free, or practically free, of biogenic amines as previously reported (Bover-Cid et al., 2000). Numerous studies, both *in vitro* and in fermented meat products, have described a reduction in biogenic amine formation by foodborne pathogens after inoculation with LAB strains (Özogul and Hamed, 2018). Among them, *L. sakei* is the most effective in reducing amine accumulation (Latorre-Moratalla et al., 2012). Bover-Cid et al. (2000) reported that the strain *L. sakei* CTC494 had a protective effect against amine formation in *fuet* (a Catalan fermented sausage), with reductions of up to 91% for putrescine, 88% for tyramine, and 74% for cadaverine. The high efficacy with which the starter culture inhibited amine formation meant it was impossible to observe any synergistic effect with cava lees.

In experiment 2, cadaverine and putrescine were also detected in the control spontaneously fermented sausages (C2), with levels of 525 and 212 mg/kg dm in the final product, respectively (Figure 1B). The formation of biogenic amines in fermented meat products can be affected by numerous variables, both intrinsic (*pH*, formulation, sausage diameter, etc.) and technological (temperature and humidity) (Vidal-Carou et al., 2014; Ruiz-Capillas and Herrero, 2019; Tabanelli, 2020). Therefore, considering that the sausages from experiments 1 and 2 shared the same formulation, diameter and processing conditions, the considerably lower levels of cadaverine and putrescine in C2 versus C1 could be attributed in part to the greater reduction of *pH* in the C2 batch (final *pH* values of 6.26 in C1 versus 5.28 in C2) (Hernández-Macias et al., 2021b). The effect of *pH* on aminogenesis is nevertheless a subject of controversy. Although an acidic *pH* is known to promote decarboxylase activity, which serves as a microbial defense mechanism against unfavorable acidic conditions, a rapid and pronounced decrease in *pH* will inhibit the growth and/or the metabolism of potentially aminogenic spoilage bacteria, consequently reducing aminogenesis (Comas-Basté et al., 2019; Ruiz-Capillas and Herrero, 2019). Tyramine was also found in this same batch, with values very similar to those found in C1 (196 mg/kg dm).

The LPE added in this experiment (E2) also had a significant aminogenesis reducing effect, 21% lower content for cadaverine and 40% for putrescine compared with the control (C2) (Figure 1B). As reported in Hernández-Macias et al. (2021b), E2 showed slightly lower levels of *Salmonella* than C2 at the end of the ripening (differences of 0.71 log_10_). Therefore, the reduction in amine levels of E2 could be due to the polyphenols from LPE, as indicated by some other studies performed on different types of meat products. For example, the addition of phenolic extracts from tea, grape, bamboo, or roses induced a significant decrease in levels of cadaverine, tyramine, histamine, and/or putrescine, which was always correlated with lower counts of spoilage bacteria in the polyphenol-enriched products (Fan et al., 2015; Wang et al., 2015; Zhang et al., 2017).

As in the case of experiment 1, the use of *L. sakei* CTC494 as starter culture, both with or without LPE, avoided the formation of the three amines. In contrast, the use of *L. sakei* BAP110 culture, also without aminogenic capacity *in vitro*, prevented the formation of putrescine and cadaverine but not totally that of tyramine (Figure 1B). Moreover, the combined use of the LPE and L. sakei BAP110 did not reduce tyramine formation more than in the control (C2 + BAP110).

It should be noted that the formation of histamine was not observed in any batch of fermented sausages. According to various studies, histamine is found in less frequency in meat products or only in low quantities. However, the occasional presence of extremely high levels of histamine has been linked to hygienically defective raw materials (European Food Safety Authority [EFSA], 2011; Comas-Basté et al., 2019).

## Conclusion

The two fermented products considered in this study showed important differences regarding the occurrence of biogenic amines. In the case of bread, only very low putrescine, cadaverine and/or spermidine contents were found, mainly proceeding from raw materials, and without significant differences depending on whether or not cava lees were added. On the contrary, the addition of cava lees or its phenolic extract in the formulation of fermented meat products has been shown to be an effective strategy to reduce the high levels of biogenic amines, such as putrescine and cadaverine, although this effect was not as effective as the use of starter cultures. In view of the obtained results on the anti-aminogenic effect in meat fermented products, it would be convenient to carry out further studies assessing a wider range of concentrations of cava lees or its phenolic extract and also to extend it to other fermented products.

## Data Availability Statement

The original contribution presented in this study are included in the article, further inquiries can be directed to the corresponding authors.

## Author Contributions

ML-M, MV-C, AJ, and SB-C: conceptualization. SH-M, NF-B, AM-G, OC-B, and ML-M: investigation. SH-M, OC-B, and ML-M: writing—original draft preparation. SH-M, NF-B, OC-B, MR-A, EL-T, ML-M, AJ, SB-C, and MV-C: writing—review and editing. MV-C, AJ, and SB-C: supervision. All authors have read and agreed to the published version of the manuscript.

## Conflict of Interest

The authors declare that the research was conducted in the absence of any commercial or financial relationships that could be construed as a potential conflict of interest.

## Publisher’s Note

All claims expressed in this article are solely those of the authors and do not necessarily represent those of their affiliated organizations, or those of the publisher, the editors and the reviewers. Any product that may be evaluated in this article, or claim that may be made by its manufacturer, is not guaranteed or endorsed by the publisher.
